# Burden of In-Hospital Admissions and Outcomes of Thoracic Outlet Compression Syndrome in the United States From 2010 to 2021

**DOI:** 10.7759/cureus.71608

**Published:** 2024-10-16

**Authors:** Fidelis E Uwumiro, Oluwatoyin Ayo-Farai, Emmanuel O Uduigwome, Stafford Nwebonyi, Emmanuel S Amadi, Oluwatobi A Faniyi, Ihunanya Kanu, Emmanuel A Babawale, Gloria Alufohai, Chukwuebuka Aguchibe, Ifeanyi Agu

**Affiliations:** 1 Internal Medicine, Prime Healthcare-SRGA (Southern Regional Georgia), Riverdale, USA; 2 Epidemiology and Public Health, Jiann-Ping Hsu College of Public Health, Georgia Southern University, Statesboro, USA; 3 Internal Medicine, Lagos University Teaching Hospital, Lagos, NGA; 4 Internal Medicine, Nnamdi Azikiwe University Teaching Hospital, Nnewi, NGA; 5 Internal Medicine, Hallel Hospital Port Harcourt, Port Harcourt, NGA; 6 Internal Medicine, Jackson State University, Jackson, USA; 7 Internal Medicine, Griffith College, Dublin, GBR; 8 Internal Medicine, Irrua Specialist Teaching Hospital, Irrua, NGA; 9 Internal Medicine, College of Medicine, University of Nigeria, Nsukka, NGA; 10 Internal Medicine, College of Medicine, Imo State University, Owerri, NGA

**Keywords:** arterial occlusive diseases, arterial thoracic outlet syndrome, average length of hospital stay, cox proportional hazards models, healthcare costs, in-hospital mortality, neurogenic thoracic outlet syndrome, readmission risk, scalenectomy, venous thromboembolism (vte)

## Abstract

Introduction

Despite advancements in medical and surgical management, thoracic outlet syndrome (TOS) remains a complex and often understudied condition with variable outcomes. This study assessed hospitalization rates and outcomes, including patient characteristics, mortality risks, and healthcare costs associated with TOS hospitalizations.

Methods

We analyzed elective and nonelective hospitalization data for TOS between 2010 and 2021 from the National Inpatient Sample (NIS) and National Readmission Databases (NEDS) and classified the data into neurogenic, venous, and arterial subtypes using the International Classification of Diseases (ICD) diagnostic and procedural codes. The primary endpoint of this study was hospital-related all-cause mortality. Secondary outcomes included hospitalization costs, length of hospital stay, in-hospital complications, and 30-day readmissions. The odds of primary and secondary outcomes were assessed using multivariate hierarchical logistic regression analysis. Cox proportional hazard models were used to assess predictors of 30-day readmission.

Results

A total of 37,174 hospitalizations for TOS were identified in the NIS datasets included in our study. Of these, 7,397 (19.9%) were for venous TOS, 3,346 (9.0%) were for arterial TOS, and 26,430 (71.1%) were for neurogenic TOS. Patients with arterial TOS were significantly older (median age: 66; interquartile range (IQR): 54-77 years) compared with venous (63 years; IQR: 50-74) or neurogenic TOS (58 years; IQR: 53-73; P < 0.001). Scalenectomy, with or without first rib resection, was performed in 18% (6,692) of TOS hospitalizations, mainly in neurogenic TOS (16.7%, 4,405 cases) compared to venous (13%, 964 cases) and arterial TOS (38.1%, 1,273 cases). The median duration of hospitalization for TOS was three days (IQR: two to six days). The mean cost of care for all TOS hospitalizations was $107,481 (standard deviation (SD): $4,158). The mean cost of hospitalization was significantly higher for vascular TOS than neurogenic TOS ($114,824 vs. $98,278; P < 0.001) and for venous TOS than arterial TOS ($119,042 vs. $110,606; P = 0.041). Overall, in-hospital mortality was 446 (1.2%). Mortality rates were significantly higher in venous TOS compared to arterial TOS (263 (59.1%) vs. 182 (40.7%); adjusted hazards ratio (AHR): 1.56; 95% confidence interval (CI): 1.26-3.56; P = 0.041). Black race (adjusted Odds ratio (aOR): 3.86, 95% CI: 8.80-16.90; P = 0.043), deep vein thrombosis (aOR: 1.68, 95% CI: 1.18-2.03; P = 0.018), previous coronary artery bypass graft (aOR: 2.37, 95% CI: 1.84-3.92; P = 0.003), pulmonary embolism (aOR: 2.63, 95% CI: 1.23-3.45; P < 0.001), and postoperative sepsis with multiorgan failure (aOR: 3.33, 95% CI: 2.13-6.40; P = 0.032) were correlated with mortality.

Conclusion

Hospitalization duration and mortality rates for TOS are generally low, though vascular TOS has a longer length of stay and higher mortality than neurogenic TOS. Mortality was significantly associated with Black race, deep vein thrombosis, previous coronary artery bypass grafting (CABG), pulmonary embolism, and postoperative septicemia.

## Introduction

Thoracic outlet syndrome (TOS) is a constellation of disorders resulting from the compression of neurovascular structures as they traverse the thoracic outlet [1]. It is classified into three main types: neurogenic TOS due to brachial plexus compression (nTOS); arterial TOS (aTOS) due to subclavian arterial compression, aneurysmal degeneration, and subsequent thromboembolic complications; and venous TOS (vTOS), which affect subclavian arteries and veins, respectively [1,2]. The term "thoracic outlet syndrome" is contentious, as compression occurs at the thoracic inlet defined by the clavicle, first rib, and the area from the axilla to the supraclavicular fossa [2]. The symptoms of TOS can range from mild to severe. Initial signs may include paleness, tingling, pain, swelling, muscle weakness, and atrophy in the upper extremities. In more severe cases, particularly in individuals with vTOS, symptoms can progress to axillo-subclavian vein thrombosis, chronic arm swelling, and persistent pain. Additional complications may include gangrene, ischemic ulcers of the fingers caused by reduced blood flow, permanent nerve damage, and pulmonary embolism [3,4].

The incidence and prevalence of TOS remain unclear, which is a challenge partly due to the lack of universally accepted diagnostic criteria and the difficulty in distinguishing normal brachial plexus compression from true TOS. The diverse causes of TOS likely lead to a broad spectrum of outcomes following both nonsurgical and surgical treatment. This variability contributes to continued debate and a shortage of prospective high-quality evidence for proper diagnosis and management [5-7]. Recent comprehensive studies on the hospitalization patterns of TOS in the United States are limited. The latest significant analysis conducted by George et al. using data from 2010 to 2015 revealed an increase in the frequency of TOS surgical procedures [8]. Concurrently, there was a decline in the incidence of both surgical complications and average hospital expenses during the study period [8]. A study on 2016-2018 transthoracic first rib resections by Issa et al. found that transthoracic first rib resection by vascular surgeons was the predominant surgical intervention for TOS, with peripheral nerve specialists conducting 10% of these surgeries [9]. Multidisciplinary care, including the application of orthoses alongside exercise, exercise alone, or a combination of exercise, various physical modalities, ergonomic modifications, and the Edgelow protocol have become important modalities for the conservative management of TOS [10].

The Edgelow protocol for TOS is a comprehensive physical therapy approach aimed at relieving symptoms and improving function by addressing compression of the nerves, arteries, or veins in the thoracic outlet. The protocol emphasizes breathing, relaxation, posture, and positioning to reduce stress and compression on the narrowed thoracic outlet. It incorporates postural correction, scapular stability exercises, and neural mobilization techniques to improve shoulder mechanics and relieve nerve irritation. Additionally, it includes targeted stretching of tight muscles such as the scalene, pectoralis minor, and upper trapezius, alongside diaphragmatic breathing techniques to reduce neck and chest tension. A key aspect of the protocol is the reliance on self-motivated home exercises between supervised therapy sessions, promoting patient engagement in their own recovery. The Edgelow protocol is often chosen for initial physical therapy consultations due to anecdotal evidence of its effectiveness in improving symptoms in TOS patients, making it a conservative yet effective option to manage pain and restore functionality without surgical intervention [11].

Existing studies on TOS are limited by sample size and practice setting, resulting in gaps in our understanding of resource utilization, hospitalization patterns, and readmission rates. This study used the most recent large national database to enhance our understanding of the nationwide burden of surgical interventions and resource use over the last decade. Additionally, we identified the rates and predictors of 30-day readmissions for TOS in the United States.

## Materials and methods

Data source

We collected data from the Healthcare Cost and Utilization Project (HCUP), the National (formerly Nationwide) Inpatient Sample (NIS), and the Nationwide Readmissions Database (NRD) for TOS hospitalizations between 2010 and 2021. Both databases are components of the HCUP, sponsored by the Agency for Healthcare Research and Quality (AHRQ). The NIS is the largest all-payer inpatient healthcare database in the United States capturing approximately 20% of discharges from all hospitals participating in HCUP. It records about seven million unweighted hospital stays annually and provides weighted national estimates of roughly 35 million hospitalizations each year. The NIS compiles a vast array of information including patient demographics (age, sex, race, and median household income by ZIP code), hospital characteristics (location, bed size, teaching status, and ownership), and in-depth clinical details from discharge abstracts, which include diagnoses and procedures coded in the International Classification of Diseases, Ninth Revision, Clinical Modification (ICD-9-CM) till October 1, 2015, and the 10th Revision (ICD-10-CM/Procedure Coding System (PCS)) thereafter. Unweighted, the NRD contains data from approximately 16.8 million discharges annually. Weighted, it estimates roughly 33.4 million discharges. Both databases record insurance status, mortality, total charges, discharge disposition, lengths of hospital stay, and detailed patient metrics such as severity, risk of mortality, and comorbidities, providing invaluable data for healthcare trend analysis and policy impact assessments [12,13].

Study cohort selection

**Figure 1:**
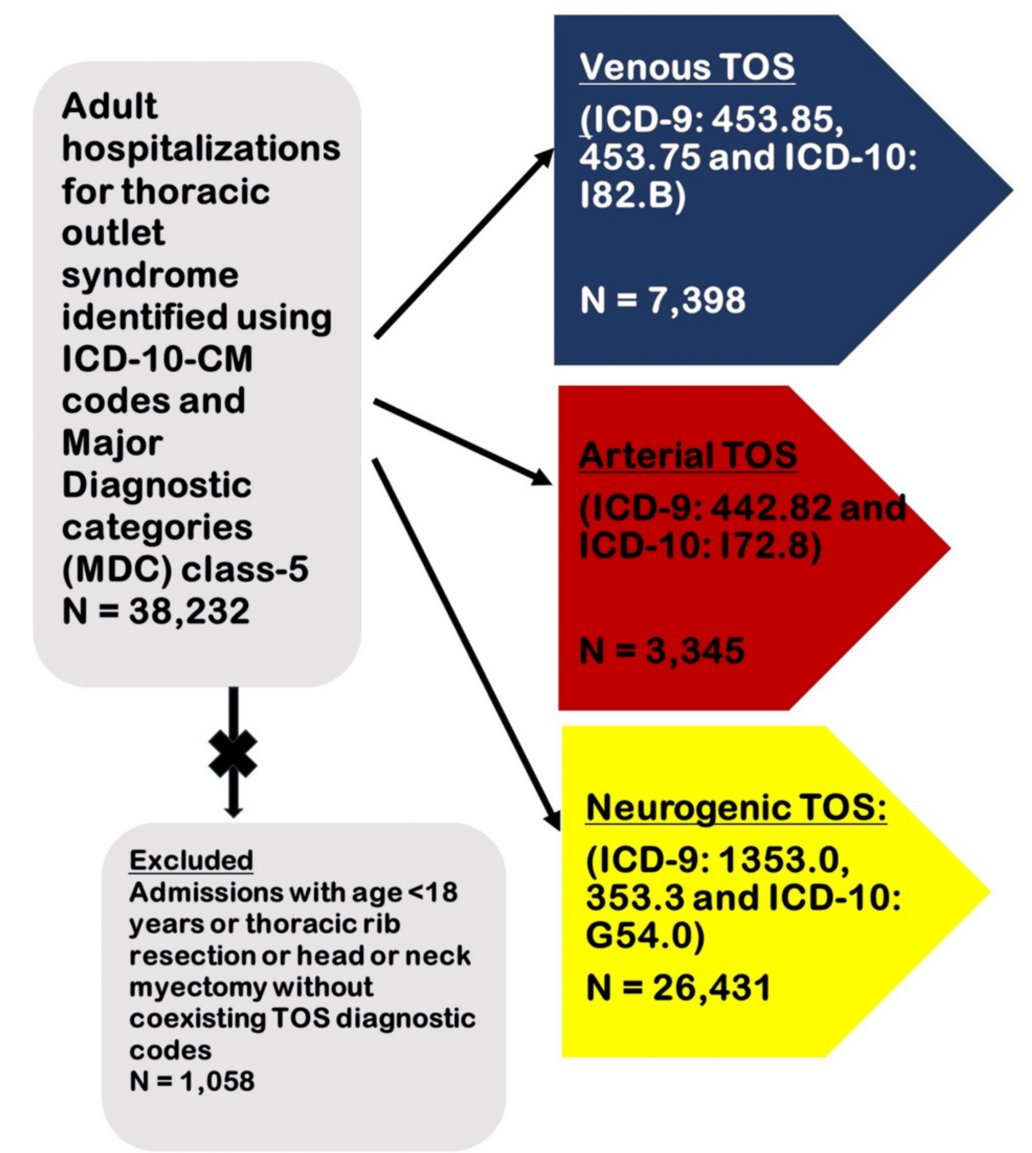
Algorithm for derivation of the study cohort ICD-10: International Classification of Diseases, Tenth Revision; NIS: Nationwide inpatient sample database; Major diagnostic category class 5: Diseases and disorders of the circulatory system; TOS: Thoracic outlet compression syndrome.

The United States transitioned to the ICD-10-CM/PCS on October 1, 2015. Accordingly, we used ICD-9-CM/PCS for the first quarter of 2015 and ICD-10-CM/PCS for subsequent hospitalizations. This ensures coding accuracy and consistency in data analysis across the study period. Consistent with previously validated methodology, hospitalizations for diseases and disorders of the cardiovascular system (major diagnostic category-5) with ICD diagnosis codes of brachial plexus lesions (ICD-9: 1353.0, 353.3 and ICD-10: G54.0), axillary/subclavian aneurysm (ICD-9: 442.82 and ICD-10: I72.8), or axillo-subclavian deep venous thrombosis (ICD-9: 453.85, 453.75 and ICD-10: I82.B) were first used to identify neurogenic, arterial, and venous TOS subtypes, respectively (Figure 1). Subsequently, ICD procedure codes used previously for rib resection (ICD-9: 77.91, 77.31, 77.41, 77.61, 77.69, 77.71, 77.79, 77.81 and ICD-10: 0PT10ZZ) and scalenectomy (ICD-9: 83.45 and ICD-10: OKB1XXX, OKB2XXX) were identified for hospitalizations in the study [8,14]. We identified thrombolysis during hospitalization among patients with vTOS using the ICD-9 (99.10) and ICD-10 codes (3E03317, 3E04317, 3E05317, 3E06317, and 3E08317). We included all adult elective or nonelective hospitalizations based on the binary "ELECTIVE" variable within the NIS dataset. Hospitalizations for thoracic rib resection or head or neck myectomy without coexisting TOS diagnostic codes were excluded. Our methodology aligns with that of a previous study [8]. The severity of illness and mortality risk at admission are assessed using the all-patient refined-diagnosis-related groups (APR-DRG) system, which classifies patients into four levels of severity, from minor to extreme, based on their primary diagnosis, age, comorbidities, and other relevant factors, to more accurately predict health care needs and outcomes [14,15].

Study outcomes

In-hospital mortality was defined in the NIS cohort using the binary variable "DIED." The duration and cost of hospitalization were defined using the NIS variables LOS and TOTCHG, respectively, which record the length of stay and associated costs for each patient. In-hospital complications including acute limb pain, limb ischemia, subclavian artery aneurysm, subclavian vein thrombosis, and post-thrombotic syndrome were identified using ICD-9 and ICD-10 codes (see Appendix). Postoperative complications for TOS across the United States have been studied in recent research [8,16].

Statistical analysis and study endpoints

All analyses were performed, and findings were presented using the weighted sample, incorporating adjustments for clustering (HOSP_NIS), weighting (DISCWT), and stratification (NIS_STRATUM) within the NIS to ensure that the results accurately reflect the broader US population [17]. To produce national estimates using NIS data from 2010 and 2011, revised discharge weights were utilized according to the 2012 HCUP NIS redesign, replacing the original discharge weights. This approach ensures that the estimates are aligned with the methodologies applied in the new NIS redesign from 2012 [18].

Sociodemographic variables were calculated as counts and percentages for categorical data. Continuous variables were presented using a median with an interquartile range or mean and standard deviations for normally and nonnormally distributed data. A hierarchical multivariate logistic regression analysis, adjusting for all variables and comorbidities listed in Table 1, was used to assess variables significantly correlated with mortality. The significance level for inclusion was set at P < 0.2. A P-value threshold of <0.2 was used in multivariate logistic regression to prevent the early exclusion of variables that may later show significant associations after adjustment. This relaxed threshold helps capture potential confounders and ensures a more thorough evaluation during model refinement. It is also useful in exploratory studies to flag variables for further investigation, even if they do not meet conventional significance levels [19].

**Table 1:** Baseline characteristics of hospitalizations for thoracic outlet syndrome Data is presented as absolute numbers (n) and percentages (%) unless otherwise specified. TOS: Thoracic outlet syndrome; DRG: Diagnosis-refined groups; CCI: Charlson comorbidity index; THC: Total hospital charges; AMI: Acute myocardial infarction; CHF: Congestive heart failure; COPD: Chronic obstructive pulmonary disease; CABG: Coronary artery bypass grafting; PCI: Percutaneous coronary intervention; IQR: Interquartile range. * P-values < 0.05 were considered statistically significant.

Variables	N (%) unless otherwise specified			
	Neurogenic TOS (n = 26,431)	Venous TOS (n = 7,398)	Arterial TOS (n = 3,345)	P*
Median age, years (IQR)	58 (53-73)	63 (50-74)	66 (54-77)	<0.001
Sex				<0.001
Female	12,132 (45.9)	3,499 (47.3)	1,776 (53.1)	
Male	14,299 (54.1)	3,898 (52.7)	1,569 (46.9)	
Race/ethnicity				<0.001
White	20,431 (77.3)	4,579 (61.9)	2,358 (70.5)	
Black	3,013 (11.4)	1,701 (23.0)	452 (13.5)	
Hispanic	1,718 (6.5)	695 (9.4)	348 (10.4)	
Asian or Pacific Islander	396 (1.5)	155 (2.1)	80 (2.4)	
Native American	132 (0.5)	37 (0.5)	20 (0.6)	
Other	740 (2.8)	229 (3.1)	90 (2.7)	
Insurance status				<0.001
Medicare	7,665 (29)	4,143 (56.0)	1,994 (59.6)	
Medicaid	2,960 (11.2)	1,250 (16.9)	442 (13.2)	
Private	14,696 (55.6)	1,753 (23.7)	803 (24.0)	
Self-pay	1,110 (4.2)	259 (3.5)	107 (3.2)	
All patient refined (DRG): risk of mortality				<0.001
Minor likelihood of dying	13,532 (51.2)	732 (9.9)	458 (13.7)	
Moderate likelihood of dying	5,180 (19.6)	1,317 (17.8)	1,080 (32.3)	
Major likelihood of dying	6,053 (22.9)	2,330 (31.5)	1,167 (34.9)	
Extreme likelihood of dying	1,665 (6.3)	3,018 (40.8)	639 (19.1)	
All patient refined (DRG): severity of illness				<0.001
Minor loss of function	132 (0.5)	44 (0.6)	64 (1.9)	
Moderate loss of function	14,088 (53.3)	592 (8.0)	1,004 (30.0)	
Major loss of function	9,938 (37.6)	2,641 (35.7)	1,495 (44.7)	
Extreme loss of function	2,299 (8.7)	4,128 (55.8)	786 (23.5)	
Charlson comorbidity index				<0.001
0	15,568 (58.9)	2,168 (29.3)	1,134 (33.9)	
1	6,396 (24.2)	2,323 (31.4)	1,184 (35.4)	
2	4,467 (16.9)	2,907 (39.3)	1,027 (30.7)	
Patient location				0.012
“Central” counties of metro areas of ≥1 million population	8,167 (30.9)	2,441 (33.0)	1,024 (30.6)	
“Fringe” counties of metro areas of ≥1 million population	7004 (26.5)	1835 (24.8)	853 (25.5)	
Counties in metro areas with 250,000–999,999 people	5233 (19.8)	1480 (20.0)	682 (20.4)	
Counties in metro areas with 50,000–249,999 people	2267 (8.5)	621 (8.4)	288 (8.6)	
Micropolitan counties	2220 (8.4)	570 (7.7)	278 (8.3)	
Not metropolitan or micropolitan counties	1533 (5.8)	444 (6.0)	224 (6.7)	
Annual median household income (quartile)				<0.001
First (0-25th)	5973 (22.6)	2375 (32.1)	967 (28.9)	
Second (26th-50th)	5788 (21.9)	1924 (26.0)	846 (25.3)	
Third (51st-75th)	6793 (25.7)	1694 (22.9)	810 (24.2)	
Fourth (76th-100th)	7903 (29.9)	1413 (19.1)	726 (21.7)	
Hospital location and teaching status				<0.001
Rural hospital	819 (3.1)	296 (4.0)	144 (4.3)	
Urban nonteaching hospital	3330 (12.6)	1339 (18.1)	532 (15.9)	
Urban teaching hospital	22281 (84.3)	5763 (77.9)	2669 (79.8)	
Hospital bed size				<0.001
Small	3515 (13.3)	1132 (15.3)	488 (14.6)	
Medium	6211 (23.5)	1916 (25.9)	846 (25.3)	
Large	16704 (63.2)	4350 (58.8)	2010 (60.1)	
Discharge quarter				<0.001
First	6423 (24.3)	1931 (26.1)	836 (25.0)	
Second	6608 (25.0)	1776 (24.0)	793 (23.7)	
Third	7110 (26.9)	1813 (24.5)	840 (25.1)	
Fourth	6291 (23.8)	1879 (25.4)	876 (26.2)	
Hospital region (%)				<0.001
Northeast	5101 (19.3)	1235 (16.7)	622 (18.6)	
Midwest	6079 (23.0)	1561 (21.1)	776 (23.2)	
South	8775 (33.2)	3144 (42.5)	1231 (36.8)	
West	6476 (24.5)	1472 (19.9)	713 (21.3)	
Weekend versus weekday admissions				<0.001
Weekend admission	4282 (16.2)	1679 (22.7)	723 (21.6)	
Nonelective admission	19400 (73.4)	6532 (88.3)	2853 (85.3)	
Elective admission	7031 (26.6)	866 (11.7)	492 (14.7)	
Comorbidities				
Old AMI	2511 (9.5)	799 (10.8)	368 (11.0)	<0.001
Old PCI	1216 (4.6)	281 (3.8)	191 (5.7)	<0.001
Old CABG	819 (3.1)	274 (3.7)	154 (4.6)	<0.001
Hypertension	7269 (27.5)	2190 (29.6)	1328 (39.7)	<0.001
Dyslipidemia	6581 (24.9)	1931 (26.1)	1157 (34.6)	<0.001
Tobacco smoking	10229 (38.7)	2382 (32.2)	1288 (38.5)	<0.001
Obese	2696 (10.2)	518 (7.0)	291 (8.7)	<0.001
CHF	4440 (16.8)	2182 (29.5)	736 (22.0)	<0.001
Sleep apnea	2114 (8.0)	577 (7.8)	304 (9.1)	0.061
Peripheral vascular disease	4097 (15.5)	784 (10.6)	696 (20.8)	<0.001
Renal disease	3489 (13.2)	2005 (27.1)	806 (24.1)	<0.001
Types 1 and 2 diabetes	4573 (17.3)	2330 (31.5)	893 (26.7)	<0.001
COPD	4758 (18.0)	1827 (24.7)	779 (23.3)	<0.001
Cerebrovascular disease	1427 (5.4)	925 (12.5)	452 (13.5)	<0.001
Rheumatoid disease	740 (2.8)	274 (3.7)	130 (3.9)	<0.001
Liver disease	846 (3.2)	525 (7.1)	425 (12.7)	<0.001
Dementia	317 (1.2)	525 (7.1)	194 (5.8)	<0.001
Cancer	1295 (4.9)	303 (4.1)	97 (2.9)	<0.001
Hemiplegia or paraplegia	555 (2.1)	422 (5.7)	94 (2.8)	<0.001
Peptic ulcer disease	211 (0.8)	229 (3.1)	37 (1.1)	<0.001
Cancer	1295 (4.9)	451 (6.1)	110 (3.3)	<0.001

Hazard ratios (HR) for both univariable and multivariable analyses, assessing the risk of 30-day readmissions, were determined using Cox proportional hazard models. These HRs were reported alongside their 95% confidence intervals (CIs) as HRs (95% CI). For the multivariable Cox proportional hazard analysis, variables that showed a significance level of <0.2 in the univariable analysis were included as covariates, and the outcomes were reported as adjusted HR (AHR) with 95% CI. The resource use burden for TOS was compared using linear logistic regression. All statistical tests were two-sided, with p-values < 0.05 deemed to reflect statistically significant associations.

Baseline illness severity and mortality risk were evaluated using the APR-DRG system, which calculates severity and mortality risk scores at admission. These scores are derived from discharge codes, factoring in primary and secondary diagnoses, age, and preexisting conditions, but excluding codes for complications developed during the hospital stay. The APR-DRG system categorizes patients into four distinct levels of functional loss and likelihood of dying (LOD): minor, moderate, major, and extreme, facilitating the assessment and adjustment of illness severity and mortality risk upon admission. The validity of the APR-DRG system has been assessed in a previous study [15]. All analyses were conducted using Stata® software, version 17MP (StataCorp LLC, College Station, TX).

## Results

Missing covariate data

Missing covariate data varied between 0.01% and 3.9%, with the "RACE" variable having the highest rate of missing data at 3.93%. On average, the rate of missing data was 0.08% per hospitalization (see Appendix). Given the minimal percentage of missing covariate data, no hospitalization was excluded for missing data.

National hospitalization volume

A total of 7,587 TOS hospitalizations were identified from the NIS datasets included in our study. When discharge and trend weights were applied to calculate national estimates, this amounted to 37,174 TOS hospitalizations in the United States during the 11-year study period. Using the national estimates of annual TOS volumes, the mean number of TOS cases annually was 3,314 (standard deviation [SD], 514). About 13,159 (35.4%) were admitted to hospitals in the southern region of the United States, whereas 6,766 (18.2%) were admitted to hospitals in the northeast, 22.4% (8,327) in the Midwest, and 21.9% (8,141) at western hospitals. The majority (22,565; 60.7%) of TOS hospitalizations were at high-volume hospitals compared to 14,609 cases (39.3%) at low-volume hospitals. About 20,112 (54.1%) were male, whereas 17,063 (45.9%) were female. The majority were white (28,736; 77.3%), Black (4,238; 11.4%), or Hispanic (2416; 6.5%) and had private (20,669; 55.6%) or Medicare insurance (10,781; 29%). Patients with arterial TOS were significantly older (median age: 66; IQR: 54-77) compared with venous (63 years; IQR: 50-74) or neurogenic TOS (58 years: IQR: 53-73; P < .001; Table 1). Table 1 summarizes illness severity, comorbidity, and other sociodemographic characteristics of the study cohort.

Outcomes

There were 446 mortalities (1.2%) overall. Of these, 298 (67%) were male, and 147 (33%) were female (P < 0.001). Mortality rates were significantly higher in venous TOS (263; 59.1%) compared to arterial TOS (182; 40.7%; P < 0.001). One death (0.2%) was recorded among hospitalizations for neurogenic TOS patients. Significant predictors of mortality in TOS hospitalizations include Black race (aOR: 3.86, 95% CI: 8.80-16.90; P = 0.043), deep vein thrombosis (aOR: 1.68, 95% CI: 1.18-2.03; P = 0.018), previous coronary artery bypass grafting (aOR: 2.37, 95% CI: 1.84-3.92; P = 0.003), pulmonary embolism (aOR: 2.63, 95% CI: 1.23-3.45; P < 0.001), and postoperative sepsis (aOR: 3.33, 95% CI: 2.13-6.40; P = 0.032), all of which significantly elevated the mortality risk.

The median duration of hospitalization for TOS was three days (IQR: 2-6 days). Approximately 510 (9.1%) hospitalizations were in the top decile for length of hospital stay (≥10 or more days). No significant difference was observed in the median LOS for neurogenic TOS compared to vascular TOS (3 vs. 4; P = 0.864). The mean cost of care for all TOS hospitalizations was $107,481 (SD, $4,158). The mean cost of hospitalization was significantly higher for vascular TOS compared to neurogenic TOS ($114,824 vs. $98,278; P < 0.001) and for venous TOS compared to arterial TOS ($119,042 vs. $110,606; P = 0.041).

Scalenectomy, with or without transthoracic first rib resection, was performed in 6,692 (18%) of TOS hospitalizations, predominantly for neurogenic TOS (4,405; 16.7%) over venous (964; 13%) and arterial TOS (1,273; 38.1%). Neurogenic TOS patients frequently reported limb pain (18,904; 71.5%), while arterial TOS was mainly linked to acute limb ischemia (1,987; 59.4%) and subclavian artery aneurysms (5.6%, 186 cases). Table 2 presents the outcomes of hospitalizations for TOS.

**Table 2:** Outcomes of hospitalizations for thoracic outlet syndrome TOS: Thoracic outlet syndrome; IQR: Interquartile range; US$: United States dollar; SD: Standard deviation; THC: Total hospital costs. * P-values < 0.05 were considered statistically significant.

Outcomes	Neurogenic TOS (N = 26,431)	Venous TOS (N = 7,398), n (%)	Arterial TOS (N = 3,345), n (%)	P*
*Primary outcome*				
Inpatient mortality, n (%), aOR (95% CI; P)	46 (0.2)	269 (3.6)	186 (5.6)	<0.001
*Secondary outcomes*				
Length of stay (days), median (IQR)	3 (2-6)	4.3 (2-7)	4 (3-6)	0.248
THC (US$), mean ± SD	98,278 ± 4,709	119,042 ± 1,289	110,606 ± 3,853	0.114
Scalenectomy ± resection of the first rib, n (%)	4,405 (16.7)	964 (13.0)	1,273 (38.1)	<0.001
Limb pain, n (%)	18,904 (71.5)	405 (5.5)	2,836 (84.8)	<0.001
Limb ischemia, n (%)	75 (0.3)	216 (2.9)	1,987 (59.4)	<0.001
Post-thrombotic syndrome, n (%)	12 (0.04)	725 (9.8)	95 (2.8)	<0.001
Aneurysm of the subclavian artery, n (%)	10 (0.04)	55 (0.7)	186 (5.6)	<0.001

Thirty-day readmissions

Approximately 36,729 (98.8%) of patients hospitalized with TOS were discharged alive, and 1,487 (4%) were readmitted within 30 days. The leading cause of readmission was TOS (471, 31.7%), with pleural effusion (177 cases, 11.9%), unspecified pneumonia (59 cases, 4%), acute post-procedural pain (74 cases, 5%), and hemothorax (48 cases, 3.2%) also being significant reasons for readmission. Other notable causes included acute and chronic embolism and thrombosis of the right subclavian vein (52 cases [3.5%] and 43 cases [2.9%], respectively), acute embolism and thrombosis of the deep veins of the right upper extremity (42 cases, 2.8%), other specified pleural conditions (43 cases, 2.9%), and type 2 diabetes with peripheral angiopathy and gangrene (38 cases, 2.6%). No significant difference was observed in the median age of patients in readmissions compared to the index hospitalizations (43 vs 45 years; P = 0.625).

After adjustment for patient and hospital covariates, moderate loss of function (LOF) (adjusted hazards ratio [AHR]: 1.27; P = 0.024), major LOF (AHR: 1.38; P = 0.012), extreme LOF (AHR: 1.59; P = 0.004), residence in the same state as hospital (AHR: 1.93; P = 0.014), deep vein thrombosis (AHR: 1.38; P = 0.036), and diabetes (AHR: 1.62; P = 0.046) were correlated with an increased likelihood of readmission (Table 3).

**Table 3:** Univariable and multivariable Cox regression models for predictors of 30-day readmissions HR: Hazards ratio; HHC: Home health care; AMA: Against medical advice; LOF: Loss of function; MI: Myocardial infarction; PCI: Percutaneous coronary intervention; CABG: Coronary artery bypass graft; MI: Myocardial infarction; PVD: Peripheral vascular disease; CHF: Congestive heart failure; CKD: Chronic kidney disease; Median household income: median household income for patient's zip code. Only variables with a significance of P-value < 0.2 on univariate Cox regression analysis were added to the multivariable regression. * P-values < 0.05 were considered statistically significant.

Variables	Univariate Cox regression		Multivariate Cox regression	
	Unadjusted HR	P-value*	Adjusted HR	P-value*
Female	1.19	0.145	1.13	0.316
Age (years)				
Age ≥ 18 and < 40	0.33	0.339	-	-
Age ≥ 40 and < 60	1.05	0.676	-	-
Age ≥ 60	1.02	0.424	-	-
No. of procedures				
0-1	2.66	<0.001	1.05	0.109
≥2	0.60	<0.001	1.00	-
Discharge disposition				
Routine discharge	0.77	0.010	0.85	0.250
Discharge to short-term	1.37	0.206	-	-
Discharge to other facilities	1.09	0.485	-	-
Discharge to HHC	1.18	0.179	0.98	0.885
Discharged AMA	2.33	0.010	2.09	0.205
Discharge quarter				
First quarter	Reference	Reference	Reference	Reference
Second quarter	1.01	0.969	-	-
Third quarter	1.04	0.758	-	-
Fourth quarter	0.94	0.675	-	-
Hospital control				
Government	Reference	Reference	Reference	Reference
Private, not-for-profit	0.94	0.688	-	-
Private, investor-owned	1.20	0.353	-	-
Insurance status				
Medicare	Reference	Reference	Reference	Reference
Medicaid	0.87	0.367	-	-
Private including HMO	0.69	0.002	-	-
Self-pay	0.90	0.720	-	-
CCI				
0	Reference	Reference	Reference	Reference
1	1.06	0.807	-	-
2	1.23	0.364	-	-
≥3	2.20	<0.001	1.21	0.534
DRG severity class				
Minor LOF	Reference	Reference	Reference	Reference
Moderate LOF	1.98	<0.001	1.27	0.024
Major LOF	1.87	<0.001	1.38	0.012
Extreme LOF	1.79	<0.001	1.59	0.004
Median household income (quartile)				
First (0-25th)	Reference	Reference	Reference	Reference
Second (26th-50th)	0.89	0.373	-	-
Third (51st-75th)	0.85	0.207	0.93	0.565
Fourth (76th-100th)	0.80	0.148	0.88	0.429
Metropolitan hospital	0.69	0.147	0.70	0.193
Teaching hospital	1.05	0.680	-	-
Admitted on a weekend	0.81	0.075	0.80	0.067
Hospital bed size				
Small	Reference	Reference	-	-
Medium	0.880	0.448	-	-
Large	0.932	0.639	-	-
Discharge quarter				
First	Reference	Reference	-	-
Second	1.01	0.969	-	-
Third	1.04	0.758	-	-
Fourth	0.94	0.675	-	-
Hospital ownership				
Government	Reference	Reference	-	-
Private, not-for-profit	0.94	0.688	-	-
Private, investor-owned	1.20	0.353	-	-
LOS				
1-5 days	1.05	0.710	-	-
5-10 days	1.12	0.263	-	-
≥ 10 days	0.88	0.198	0.95	0.706
Resident of the same state as a hospital	1.98	0.005	1.93	0.014
Dyslipidemia	0.95	0.617	-	-
Previous MI	1.05	0.699	-	-
Previous PCI	1.18	0.202	-	-
Previous CABG	0.99	0.937	-	-
Old pacemaker	1.24	0.397	-	-
COPD	1.47	0.005	1.11	0.472
Carotid artery disease	1.79	0.776		
Old stroke	1.46	0.006	1.22	0.163
Hypertension	0.54	<0.001	0.69	0.118
PVD	1.57	<0.001	1.16	0.265
Hypothyroidism	1.57	<0.001	1.00	
DM type 1 and type 2	1.90	0.141	1.62	0.046
Obesity	0.76	0.097	0.74	0.079
CHF	1.48	0.002	0.99	0.990
CKD	1.77	<0.001	1.19	0.213
Liver disease	1.41	0.112	1.33	0.217
Smoking	1.04	0.920	-	-
Anemia	1.13	0.400	-	-
Alcohol use disorder	0.77	0.201	-	-
Sepsis	1.19	0.363	-	-
Depression	2.66	0.131	1.42	0.058
Deep venous thrombosis	1.68	0.147	1.38	0.036
Pulmonary embolus	2.15	0.200	-	-

## Discussion

Demographic and hospital outcomes

The index study highlights significant ethnic and racial disparities in the prevalence and healthcare use of TOS. The overall mortality rate was relatively low at 1.2%; however, there were notable differences among the TOS types. Specifically, venous TOS had a significantly higher mortality rate than arterial and neurogenic TOS. The arterial subtype of vascular TOS can cause intimal damage to the subclavian artery, potentially resulting in aneurysm formation and limb-threatening ischemia. Similarly, the venous subtype can cause intimal damage, leading to thromboembolic events [20]. Some studies have reported no mortality in hospitalized patients with TOS despite complications and morbidity [8,21-23].

The demographic distribution of TOS in our study is consistent with that of prior research, which indicated that TOS is more commonly diagnosed in teenage and young adult populations, thereby affecting productivity due to disability and medical costs. This prevalence is partly due to increased physical activity and repetitive arm and shoulder movements, which are typical in these age groups, and can contribute to TOS development. Activities such as playing sports, performing certain types of physical labor, or even prolonged postures related to studying and computer use can increase the risk of TOS in teenagers and young adults. Additionally, anatomical variations and congenital factors that predispose individuals to TOS are often identified in this younger population. However, our findings on higher disease severity in men and disparities among ethnic groups provide new insights into potential sex or racial predispositions that warrant further investigation.

Other resource use outcomes of the index study, such as length of hospital stay and total hospital charges, did not show significant differences among the TOS subtypes. This finding can be attributed to several factors. Standardized treatment protocols and overlapping management strategies for different TOS subtypes result in comparable resource utilization. The severity and complications of TOS, which require comparable medical interventions, also contribute to this uniformity. In addition, healthcare system factors, such as hospital policies and insurance reimbursement rates, play a role in standardizing costs and lengths of stay [24]. Finally, integrated multidisciplinary care approaches may ensure consistent outcomes across all TOS subtypes, as observed in other patient groups [25].

The arterial subtype of TOS requires the highest rate of surgical interventions, such as scalenectomy or first rib resection, compared with neurogenic and venous TOS because of its greater severity and anatomical compression of the subclavian artery, which leads to significant vascular complications. This compression results in a higher prevalence of limb pain and ischemia because it directly impacts blood flow to the limb, causing pain, numbness, and tissue damage. While neurogenic TOS affects nerves and venous TOS affects veins, leading to less severe symptoms such as swelling or muscle weakness, arterial TOS’s acute vascular compromise necessitates more aggressive treatment [26]. Medical protocols for arterial TOS often prioritize surgical intervention to prevent serious complications. This explains the higher rates of these procedures compared to the other subtypes, which might first attempt conservative treatments such as physical therapy [27]. As expected, post-thrombotic syndrome is most commonly observed in venous TOS, whereas subclavian aneurysms are significantly more common in arterial TOS.

Thirty-day readmission for TOS

To the best of our knowledge, this study is one of the few to evaluate 30-day readmission rates for TOS. Our findings showed that TOS-specific readmissions accounted for nearly 32% of all readmissions within 30 days of initial discharge. Individuals with TOS who experience significant functional loss, have a history of thromboembolic events, or have a substantial comorbidity burden are more likely to be readmitted within this period. Additionally, patients who undergo scalenectomy with rib resection have a higher risk of reoperation and readmission than those who undergo scalenectomy alone [28]. Lower body mass index, preoperatively contaminated wounds, inpatient procedures, and longer operative time have also been independently associated with reoperation [29,30]. Patients who underwent first rib resection for venous TOS were more likely to require blood transfusion. Additionally, surgical procedures performed by surgeons whose specialty was not among the top three most common for performing first rib resections had a 40% longer operation time and significantly higher odds of needing transfusion [31].

These findings show that postoperative care and follow-up are critical for patients with TOS due to their high readmission rates of nearly 32% within 30 days. Patients with significant functional loss, history of thromboembolic events, or significant comorbidities require tailored postoperative care to reduce readmission. The increased risk of reoperation and readmission for scalenectomy with rib resection highlights the need for careful surgical planning and patient selection. Preoperative optimization, such as wound care and reducing operative time, is essential. Surgeon expertise significantly affects outcomes, emphasizing the importance of referral to specialized centers. Additionally, a higher likelihood of blood transfusion in patients with venous TOS undergoing first rib resection necessitates preoperative planning and patient counseling. A multidisciplinary approach involving careful assessment, meticulous surgical technique, and robust postoperative care is crucial for improving outcomes in TOS patients.

Limitations

As with other studies based on billing data, this study has some limitations. The retrospective nature of the database limits our ability to establish causality. As national databases do not record individual patients, it is impossible to track individual patient outcomes during the study period. Additionally, due to the low prevalence of TOS, many hospitalizations in this study likely involved the same individuals who were readmitted multiple times. The ambiguity of the ICD-10 codes for TOS introduces potential coding inaccuracies. Furthermore, the lack of detailed clinical data, such as imaging results and specific surgical details like operating time and anesthesia duration, limits our ability to comprehensively analyze the effectiveness of treatments and operative predictors of hospital stay. Despite these limitations, this index study provides valuable insights into this important clinical issue. Future prospective studies are needed to validate these findings and explore the long-term outcomes of different TOS treatments to enhance our understanding of posttreatment quality of life. Additionally, investigation of genetic, occupational, and lifestyle risk factors associated with TOS could improve preventive strategies and early diagnosis.

## Conclusions

The mean length of hospital stay and mortality rate for TOS remain relatively low, with vascular TOS having a longer length of stay and higher mortality rate than neurogenic subtypes. The predictors of increased mortality included race, deep vein thrombosis, previous CABG, pulmonary embolism, and postoperative sepsis. Approximately 4% of patients with TOS are readmitted within 30 days due to functional loss, signs of thromboembolism, and comorbidity. Patients undergoing scalenectomy with rib resection are also more likely to be readmitted. The findings indicate a broad range of TOS presentations and the possibility of further evolution in surgical planning, patient selection, and postoperative care. The findings of this study also highlight the importance of preoperative optimization and the significant impact of surgeon expertise on patient outcomes. Future prospective studies are necessary to validate these results, explore long-term outcomes, and improve management approaches and early diagnosis. This will ultimately enhance the quality of life of TOS patients and optimize healthcare resource utilization.

## Appendices

**Table 4:** ICD-10 diagnostic and procedural codes used in the study ICD: International Classification of Diseases; MDC: Major classification of diseases; DVT: Deep vein thrombosis; MI: Myocardial infarction; PCI: Percutaneous coronary intervention; CABG: Coronary artery bypass grafting.

Description	ICD-9/ICD-10 codes
Vascular thoracic outlet syndrome	G54.0, 353
MDC-5	Hospitalizations for cardiovascular diseases and disorders
Limb pain	729.X, M79.601, M79.602, M79.603, M79.621, M79.622, M79.629, M79.631, M79.632, M79.639, M79.641, M79.642, M79.643, M79.644, M79.645, M79.646
Pulmonary embolism	415.X, I26.01, I26.02, I26.09, I26.90, I26.92, I26.93, I26.94, I26.99, I26
Sepsis	995.X, A419, A40, A41
Subclavian DVT	453.85, 453.75, I82.B11, I82.B12, I82.B13, I82.B19
Subclavian artery aneurysm	442.82, I72.8
Post-thrombotic syndrome	459.X, I87.009
Dyslipidemia	272.X, E785
Hypertension	401, 405, I10
Obesity	278.X, E669
Tobacco use	305.1, V15.82, Z720, F17.200, F17.201, F17.203, F17.208, F17.209, F17.210, F17.211, F17.213, F17.218, F17.219, F17.220, F17.290, F17.299, Z87.891
Alcohol use disorder	291.X, F10
Old DVT	453.5X, I82
Sleep apnea	780.57., G4730, G4733, G4739
Old MI	I252
Old PCI	Z955
Old CABG	Z951
Resection of the 1st rib	77.91, 77.31, 77.41, 77.61, 77.69, 77.71, 77.79, 77.81., 0PT10ZZ
Scalenectomy	83.45., 01N30ZZ, 01N33ZZ, 01N34ZZ

**Table 5:** Missing covariate data

Variables	Percent missing
Mortality	0.09
Discharge disposition	0.09
Elective vs. nonelective admission	0.36
Patient location	0.36
Race	3.93
Hospital costs	1.25
Transferred in	0.63
Transferred out	0.09
Median annual income quartiles	1.88
